# Comparisons of *Staphylococcus aureus* infection and other outcomes between users of angiotensin-converting-enzyme inhibitors and angiotensin II receptor blockers: lessons for COVID-19 from a nationwide cohort study

**DOI:** 10.12688/wellcomeopenres.15873.1

**Published:** 2020-04-27

**Authors:** Patrick Bidulka, Masao Iwagami, Kathryn E. Mansfield, Fotini Kalogirou, Angel Y. S. Wong, Ian J. Douglas, Liam Smeeth, Charlotte Summers, Laurie A. Tomlinson

**Affiliations:** 1 Department of Non-Communicable Disease Epidemiology, London School of Hygiene and Tropical Medicine, London, UK; 2 Department of Health Services Research, Faculty of Medicine, University of Tsukuba, Ibaraki, Japan; 3 Department of Medicine, Addenbrooke's Hospital, University of Cambridge, Cambridge, UK

**Keywords:** Angiotensin converting enzyme inhibitors, Angiotensin receptor blockers, Staphylococcus aureus, Sepsis, infection, COVID-19

## Abstract

**Background:** Mice receiving angiotensin converting enzyme inhibitor (ACEI) drugs show increased susceptibility to infection by *Staphylococcus aureus *( *S. aureus*). We sought to investigate whether humans using ACEI were at increased risk of *S. aureus* infection, comparing them to users of Angiotensin II Receptor Blockers (ARB) with multiple control outcomes to assess the potential for residual confounding.

**Methods:** Using the UK Clinical Practice Research Datalink linked to Hospital Episode Statistics between 1997 and 2017, we identified adults starting ACEI or ARB (as an active comparator drug). We regarded prescription of ACEI or ARB as time-dependent exposure and used a Cox regression model to compare incidence of first hospitalisation with infection due to *S. aureus* in periods with ACEI to periods with ARB prescriptions. We repeated the analysis using control outcomes that we did not expect to be associated with use of ACEI versus ARB (Gram-negative sepsis, hip fracture and herpes zoster) and one that we did (dry cough).

**Results:** We identified 445,341 new users of ACEI (mean age 64.0±14.0, male 51.7%) and 41,824 new users of ARB (mean age 64.1±14.0, male 45.5%). The fully adjusted hazard ratio for *S. aureus* infection (ACEI vs. ARB) was 1.18 (95% CI 1.10–1.27), consistent across sensitivity analyses. However, we also found associations with all control outcomes; rates of Gram-negative sepsis, hip fracture and dry cough were also increased during periods of time treated with ACEI compared to ARB while herpes zoster was more common during time treated with ARB.

**Conclusions:** Our results suggest that although ARB users appear an ideal control for analyses of ACEI effects, there is residual confounding even after multivariable adjustment. This has implications for observational analyses comparing users of these drug classes, in particular the effect of these drugs in relation to COVID-19 infection.

## Introduction

Angiotensin converting enzyme inhibitor (ACEI) drugs and angiotensin II receptor blockers (ARB) are commonly used drugs for the treatment of hypertension, proteinuric kidney diseases and heart failure ^1– 4^. The drugs block activation of different parts of the renin-angiotensin system, a hormone system that regulates fluid and electrolyte balance, and blood pressure ^5^. Despite their similar clinical uses, the separate mechanisms of action of the drug classes means that they may result in different effects on other biological systems. For example, in animal models, angiotensin converting enzyme (ACE) has a role in neutrophil antibacterial defences and this effect is independent of the angiotensin II receptor where ARB drugs act. Treatment of mice with ACEI leads to increased susceptibility to infection with *Staphylococcus aureus* ( *S. aureus*) ^6^. These findings suggest that humans taking ACEI could be at increased risk of *S. aureus* infection compared to users of ARB, but this result has not been examined in clinical studies.

The similar indications for ACEI and ARB mean that they could be ideal comparator groups in observational analyses of drug effects, reducing the potential confounding that can occur when comparing drug classes which are prescribed for different indications. Users of ACEI have been compared to ARB users in a number of important epidemiological studies, some of which have proposed a causal association between ACEI use and adverse outcomes ^7– 10^. Using anonymised primary health care records, we sought to compare rates of *S. aureus* infection between users of ACEI and ARB, including Gram-negative infections as a predefined negative control where we did not expect to see an association based on previous research in animals.

During this analysis, the pandemic of coronavirus disease 2019 (COVID-19) due to the severe acute respiratory syndrome coronavirus 2 (SARS-CoV-2) began. SARS-CoV-2 uses the ACE2 protein, a counter-regulatory component of the renin-angiotensin system, to enter alveolar epithelial cells in the lungs ^11^. Early reports suggested that people with hypertension, chronic kidney disease, cardiovascular disease, and diabetes were at higher risk for severe outcomes from COVID-19 than people without these comorbidities ^12^. It has been proposed that use of drugs affecting the renin-angiotensin system could explain this increased risk via effects on the ACE2 enzyme ^13^. However, evidence for a possible benefit from these drugs is sufficiently strong that a clinical trial has been initiated using an ARB, Losartan, as a treatment for COVID-19 ^14^.

Given the intense interest in this topic, it is likely that analyses of COVID-19 outcomes in relation to use of ACEI and ARB will be repeatedly investigated. We have previously discussed the epidemiological difficulty of comparing users of these drugs to non-users ^15^. Given the interest in potential differential effects of ACEI and ARB use in relation to COVID-19, we sought to investigate the possibility of residual confounding between users of these drug classes using a large, high-quality data source where multiple sensitivity analyses can be undertaken. Therefore, in addition to our original research question, we undertook analyses of further outcomes to explore the potential limitations of analyses between these two drug classes to inform future work regarding COVID-19.

## Methods

### Data source

The Clinical Practice Research Datalink (CPRD) is an observational data and interventional research service provided by the National Health Service (NHS). Nearly 700 general practices have contributed data meeting quality control standards to CPRD, representing nearly 7% of the UK population ^16^. The database includes the following data: patient demographics; coded diagnoses (Read codes); prescriptions; laboratory test results; and referrals recorded by general practitioners (GPs). In addition, the CPRD is linked to inpatient Hospital Episode Statistics (HES) in around 400 general practices, accounting for 75% of general practices in CPRD in England. HES contains details of all admissions to NHS hospitals in England since 1997 ^17^, and consists of primary and subsidiary diagnoses recorded during admission using the 10th revision of International Classification of Disease (ICD-10) codes ^18^. This study was approved by the LSHTM Research Ethics Committee (reference 6536) and the Independent Scientific Advisory Committee, which oversees research involving CPRD data (Protocol 18_021R). Informed consent was not required because data are anonymised for research purposes.

### Study population

We identified new users of ACEI or ARB (i.e. those without any previous prescriptions in the database), aged ≥18 years and registered in HES-linked CPRD for ≥1 year, between April 1997 and March 2017. We excluded people starting ACEI and ARB on the same day. Once individuals were included into the study cohort, they were followed up until the first incidence of an outcome or the end of CPRD eligibility, including death, change of general practice, last data collection from the general practice, or 31 ^st^ March 2017 (whichever occurred first).

### Exposure definition

We considered prescription of ACEI or ARB as time-dependent exposure. **Supplementary Figure 1** ( *Extended data* ^19^) is a graphical representation of exposure definition. First, we identified periods with ACEI and ARB prescription separately in individuals. The duration of each prescription was estimated by dividing the total number of tablets prescribed by the number of tablets to be taken each day (daily dose). When the daily dose or total number of tablets was missing (around 8.7% of the records), we imputed the median prescription duration (28 days). We assumed that people were continuously exposed to ACEI or ARB if there were no gaps of more than 60 days between the end of one prescription and the start of the next (to allow potential medication stockpiling or prescribing in secondary care). If there was no subsequent prescription of ACEI or ARB, we considered people could be influenced by the effect of the drug until 60 days after the end of the prescription. Thus, each episode of ACEI or ARB treatment started at the first prescription (as a new treatment episode) and continued until 60 days after a break in continuous prescribing of 60 days (or more). An individual could contribute multiple episodes of ACEI or ARB treatment during follow-up. Next, we classified individual participants’ follow-up time into: “period with ACEI only,” “period with ARB only,” “period with both ACEI and ARB,” and “period without ACEI or ARB” ( **Supplementary Figure 1**, *Extended data* ^19^). In the main analysis, we compared “period with ACEI only” and “period with ARB only.”

### Outcome definition

The primary outcome of interest was *S. aureus* infection identified during hospital admissions (HES) and recorded as ICD-10 codes A41.0 (Sepsis due to *Staphylococcus aureus*) and B95.6 ( *Staphylococcus aureus* as the cause of diseases classified to other chapters). We did not include outpatient diagnoses of *S. aureus* infection in the CPRD, because this could represent results of microbiological swabs not associated with clinically significant infection. In the main analysis, we defined the primary outcome using the codes of interest in any episode (a single period of care under one consultant team) within a spell (a patient’s entire stay in hospital) in any diagnostic position in HES (up to 20 diagnoses are recorded in order of priority for each episode of care).

In addition to the primary outcome, we included a number of negative control outcomes, where we did not expect to see an increased risk associated with either ACEI or ARB use. For these outcomes, any difference in risk between the groups is therefore likely to indicate unadjusted confounding. The first was pre-specified, Gram-negative sepsis (defined using ICD-10 code A41.5 in any diagnostic position in HES in any episode of any spell). *Post-hoc*, after the outbreak of COVID-19, we added additional outcomes to further explore potential residual confounding. Therefore, we also compared risk of hip fracture (defined using ICD-10 code S72 in any diagnostic position in HES in any episode of any spell), and the risk of development of herpes zoster infection (defined using both primary care Read codes and ICD-10 code B02 in HES in any diagnostic position in HES in any episode in any spell). Finally, to ensure the robustness of our comparisons we added dry cough (defined using primary care Read codes) as a positive control outcome, where we expected to see an association with ACEI use. All outcome and covariate codes used in primary care records are available for as *Underlying data* ^20^.

### Covariate definitions

Our covariates were defined *a priori*, based on knowledge of clinical factors that might influence choice of prescription of ACEI or ARB. In the main analyses, in addition to age (<55, 55–64, 65–74, 75–84, and ≥85 years), sex, and calendar period (1997–2001, 2002–2006, 2007–2011, and 2012–2016), we considered kidney function and comorbidity diagnoses of hypertension ^1^, heart failure ^2^, diabetes ^3^, proteinuria ^3^, myocardial infarction ^4^ and renal replacement therapy (RRT) as time-updated confounding factors in the association between ACEI (vs ARB) and infection due to *S. aureus*. We determined kidney function by calculating estimated glomerular filtration rate (eGFR) from serum creatinine records in the CPRD using the Chronic Kidney Disease Epidemiology Collaboration equation ^21^, and categorized this as eGFR ≥60, 45–59, 30–44, and <30 mL/min/1.73m ^2^. We used a last-carried-forward method ^22^, meaning that eGFR was initially defined using the serum creatinine result that marked entry to the study and updated at each subsequent creatinine result, so that eGFR was always defined by the single most recent creatinine result. In the main analysis we grouped individuals without any serum creatinine measurement prior to the cohort entry into the eGFR ≥60 mL/min/1.73m ^2^ category ^23^.

### Statistical analysis

We first compared individual characteristics (age category, sex, calendar period, kidney function category, and diagnoses of hypertension, heart failure, diabetes, proteinuria, myocardial infarction, and RRT) between new users of ACEI and ARB, using chi-square tests. Because a proportion of new users of ACEI and ARB switched to the other drug during follow-up, we also determined time-updated characteristics defined by periods prescribed ACEI or ARB in which the numerator was the length of time with each covariate status (e.g. coded as diabetic) and the denominator was the total length of ACEI and ARB prescription in the study population.

We estimated the crude rate of the first incidence of the outcome (i.e. hospitalisation with infection due to *S. aureus*) after cohort-entry in periods with and without ACEI and ARB prescription. We used multivariable time-dependent Cox regression analysis to estimate hazard ratios for the first incidence of each outcome, comparing periods of ACEI and ARB use and adjusting for the pre-specified, time-updated confounding factors. All statistical analyses were conducted using Stata 15 software (Stata Corp, Texas).

### Sensitivity and subgroup analyses

For the primary analysis only, to test the robustness of our findings for the definitions used we undertook a number of sensitivity analyses. First, we included “period with both ACEI and ARB” into “period with ACEI only.” Secondly, we examined our definition of ACEI and ARB exposure by changing the 60-day duration of periods between prescriptions, and washout periods to 30 and 90 days. Thirdly, we conducted an analysis including multiple covariates related to risk of infection which we did not include in the main analysis as we did not anticipate they would influence the choice of ACEI or ARB and therefore be true confounders: diagnoses of cancer, rheumatoid arthritis, systematic lupus erythematosus, inflammatory bowel disease, chronic liver disease, chronic pulmonary disease, human immunodeficiency virus infection and acquired immune deficiency syndrome. We also included prescription of other antihypertensives (beta blockers, calcium channel blockers, and diuretics), statins, oral corticosteroids, and life-style factors (smoking status, alcohol status and body mass index). Conditions were considered present if recorded in CPRD prior to cohort entry and medications were included if prescribed in the year prior to cohort entry. Information on life-style factors was based on that recorded at the closest time point to the cohort entry and not time-updated. Fourth, we repeated the main analysis excluding individuals with no serum creatinine measurements recorded prior to the cohort entry. Finally, we examined our outcome definitions in several ways. We defined the outcome using the ICD-10 codes of interest recorded only during the first episode of a spell, which are more likely to suggest community-acquired *S. aureus* infection. We then repeated the main analysis including additional non-specific ICD-10 codes indicating *Staphylococcal* infection, including A49.0 (Staphylococcal infection, unspecified site), G00.3 (Staphylococcal meningitis), J15.2 (Pneumonia due to Staphylococcus), and M00.0 (Staphylococcal arthritis and polyarthritis). Finally, we included as our outcome only the severest form of *S. aureus* infection, based on ** ICD-10 code ** A41.0 (Sepsis due to *Staphylococcus aureus*).

We also conducted pre-planned subgroup analyses to investigate potential approaches to mitigating residual confounding. To examine whether the findings were affected by changes in the recording of *S. aureus* codes ** over time, we estimated the adjusted hazard ratio separately by category of calendar year (1997–2001, 2002–2006, 2007–2011, and 2012–2016). Finally, we stratified results by previous smoking status to explore whether categorisation of ex-smokers as non-smokers in the GP record was a potential source of information bias.

## Results

**Figure 1.**
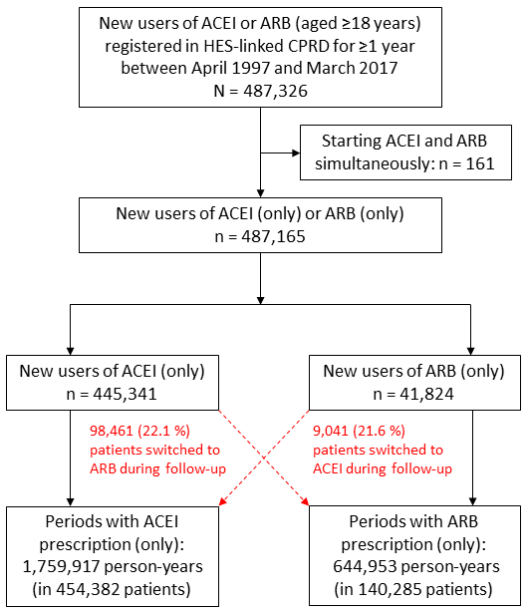
Flowchart diagram of identification of participants in the study cohort.

We identified 487,326 new users of ACEI or ARB (aged ≥18 years) registered in HES-linked CPRD for ≥1 year between April 1997 and March 2017 ( Figure 1). After excluding 161 individuals prescribed ACEI and ARB simultaneously, there were 487,165 eligible individuals, including 445,341 new users of ACEI (mean age 64.0±14.0, male 51.7%) and 41,824 new users of ARBs (mean age 64.1±14.0, male 45.5%). At cohort entry, new users of ACEI were more likely to have diabetes, myocardial infarction, heart failure, and proteinuria, whereas new users of ARB were more likely to have hypertension ( Table 1).

**Table 1.** Characteristics of the study participants at cohort entry.

	New users of ACEIs N = 445,341 n (%)	New users of ARBs N = 41,824 n (%)
**Age category (years)**		
<55	122,148 (27.4)	10,889 (26.0)
55–64	106,125 (23.8)	10,061 (24.1)
65–74	109,317 (24.6)	10,860 (26.0)
75–84	82,473 (18.5)	7,931 (19.0)
≥85	25,278 (5.7)	2,083 (5.0)
**Sex**		
Women	215,043 (48.3)	22,779 (54.5)
**Year**		
1997–2001	73,702 (16.6)	7,159 (17.1)
2002–2006	168,105 (37.8)	22,430 (53.6)
2007–2011	138,439 (31.1)	7,321 (17.5)
2012–2016	65,095 (14.6)	4,914 (11.8)
**Hypertension**	297,625 (66.8)	31,240 (74.7)
**Diabetes**	75,762 (17.0)	4,634 (11.1)
**Myocardial infarction**	40,233 (9.0)	1,621 (3.9)
**Heart failure**	23,243 (5.2)	1,061 (2.5)
**Proteinuria diagnosis**	5,925 (1.3)	336 (0.8)
**Kidney function:**		
Not measured	82,755 (18.6)	10,342 (24.7)
eGFR ≥60 mL/min/1.73m ^2^	291,925 (65.6)	24,447 (58.5)
eGFR 45-59 mL/min/1.73m ^2^	51,939 (11.7)	5,037 (12.0)
eGFR 30-44 mL/min/1.73m ^2^	15,254 (3.4)	1,432 (3.4)
eGFR <30 mL/min/1.73m ^2^	2,828 (0.6)	396 (1.0)
On renal replacement therapy	640 (0.1)	170 (0.4)

ACEI = angiotensin-converting-enzyme inhibitor, ARB = angiotensin II receptor antagonist, eGFR = estimated glomerular filtration rate.

**Table 2.** Crude rate and unadjusted and adjusted hazard ratios for the incidence of each outcome among new users of ACEI or ARB (N = 487,165).

				Crude rates/1000 PY (95% CI) for outcomes in ACEI users (defined in different ways) to ARB users, and HR (95% CI) comparing incidence rates of outcomes in ACEI to ARB users			
Outcome	Number of events	Overall incidence rate/1000 PY (95% CI)	Measure	Period with ARB prescription	Period with ACEI prescription	Period without ACEI or ARB prescription	Period with both ACEI and ARB prescriptions
**Primary outcome**							
*S. aureus* infection	6,438	2.13 (2.08 – 2.19)	Crude rate	1.56 (1.47 – 1.66)	1.95 (1.88 – 2.02)	3.33 (3.18 – 3.48)	2.47 (2.01 – 3.05)
			Unadjusted HR	1 (reference)	1.26 (1.17 – 1.35)	2.19 (2.03 – 2.36)	1.57 (1.26 – 1.95)
			Adjusted * HR	**1 (reference)**	**1.18 (1.10 – 1.27)**	1.90 (1.75 – 2.05)	1.40 (1.12 – 1.74)
**Negative control outcomes**							
Gram-negative sepsis	1,882	0.62 (0.59 – 0.65)	Crude rate	0.51 (0.45 – 0.56)	0.54 (0.51 – 0.57)	1.02 (0.94 – 1.11)	0.34 (0.19 – 0.59)
			Unadjusted HR	1 (reference)	1.17 (1.03 – 1.32)	2.11 (1.84 – 2.41)	0.72 (0.40 – 1.28)
			Adjusted * HR	**1 (reference)**	**1.16 (1.02 – 1.32)**	1.79 (1.56 – 2.06)	0.65 (0.37 – 1.16)
Herpes zoster infection	22,434	7.65 (7.55 – 7.75)	Crude rate	8.40 (8.17 – 8.63)	7.23 (7.10 – 7.36)	8.09 (7.86 – 8.33)	8.35 (7.45 – 9.37)
			Unadjusted HR	1 (reference)	0.87 (0.84 – 0.90)	0.97 (0.93 – 1.01)	1.01 (0.90 – 1.14)
			Adjusted * HR	**1 (reference)**	**0.91 (0.88 – 0.94)**	0.96 (0.93 – 1.00)	1.04 (0.92 – 1.17)
Hip fracture	12,088	4.03 (3.95 – 4.10)	Crude rate	3.23 (3.10 – 3.38)	3.60 (3.51 – 3.69)	6.33 (6.12 – 6.54)	2.59 (2.10 – 3.17)
			Unadjusted HR	1 (reference)	1.17 (1.12 – 1.23)	2.01 (1.90 – 2.12)	0.84 (0.68 – 1.03)
			Adjusted * HR	**1 (reference)**	**1.31 (1.25 – 1.38)**	1.63 (1.54 – 1.72)	0.95 (0.77 – 1.17)
**Positive control outcome**							
Dry cough	11,697	3.94 (3.87 – 4.01)	Crude rate	2.62 (2.49 – 2.75)	4.96 (4.85 – 5.06)	1.93 (1.82 – 2.05)	9.08 (8.12 – 10.14)
			Unadjusted HR	1 (reference)	1.51 (1.43 – 1.59)	0.75 (0.70 – 0.81)	2.22 (1.96 – 2.50)
			Adjusted * HR	**1 (reference)**	**1.59 (1.51 – 1.68)**	0.79 (0.73 – 0.86)	2.19 (1.94 – 2.48)

ACEI = angiotensin-converting-enzyme inhibitor, ARB = angiotensin II receptor antagonist, CI = confidence interval, HR = hazard ratio.

* Adjusted for age (<55, 55-64, 65-74, 75-84, and ≥85 years), sex, year (1997-2001, 2002-2006, 2007-2011, 2012-2016), kidney function (eGFR ≥60 mL/min/1.73m ^2^ or unmeasured, eGFR 45-59 mL/min/1.73m ^2^, eGFR 30-44 mL/min/1.73m ^2^, eGFR <30 mL/min/1.73m ^2^, or renal replacement therapy), diagnoses of hypertension, diabetes, myocardial infarction, heart failure, and proteinuria (all covariates were time-updated).

During follow-up, 98,461 (22.1 % of 445,341) of new users of ACEI switched to ARB, and 9,041 (21.6 % of 41,824) of new users of ARB switched to ACEI ( Figure 1). Consequently, there were 1,759,917 person-years of ACEI prescriptions, and 644,953 and person-years of ARB prescriptions. The patterns of participant characteristics between periods of ACEI and ARB prescriptions during follow-up were similar to, but generally smaller than, the differences between new users of ACEI and ARB at cohort entry ( **Supplementary Table 1,** *Extended data* ^19^).

In the primary analysis, there were 3,430 and 1,007 first hospitalisations with infection due to *S. aureus* in periods with ACEI and ARB, providing the crude incidence rates/1000 person years (95% CI) of 1.95 (1.88 – 2.02) and 1.56 (1.47 – 1.66), respectively ( Table 2).

**Figure 2.**
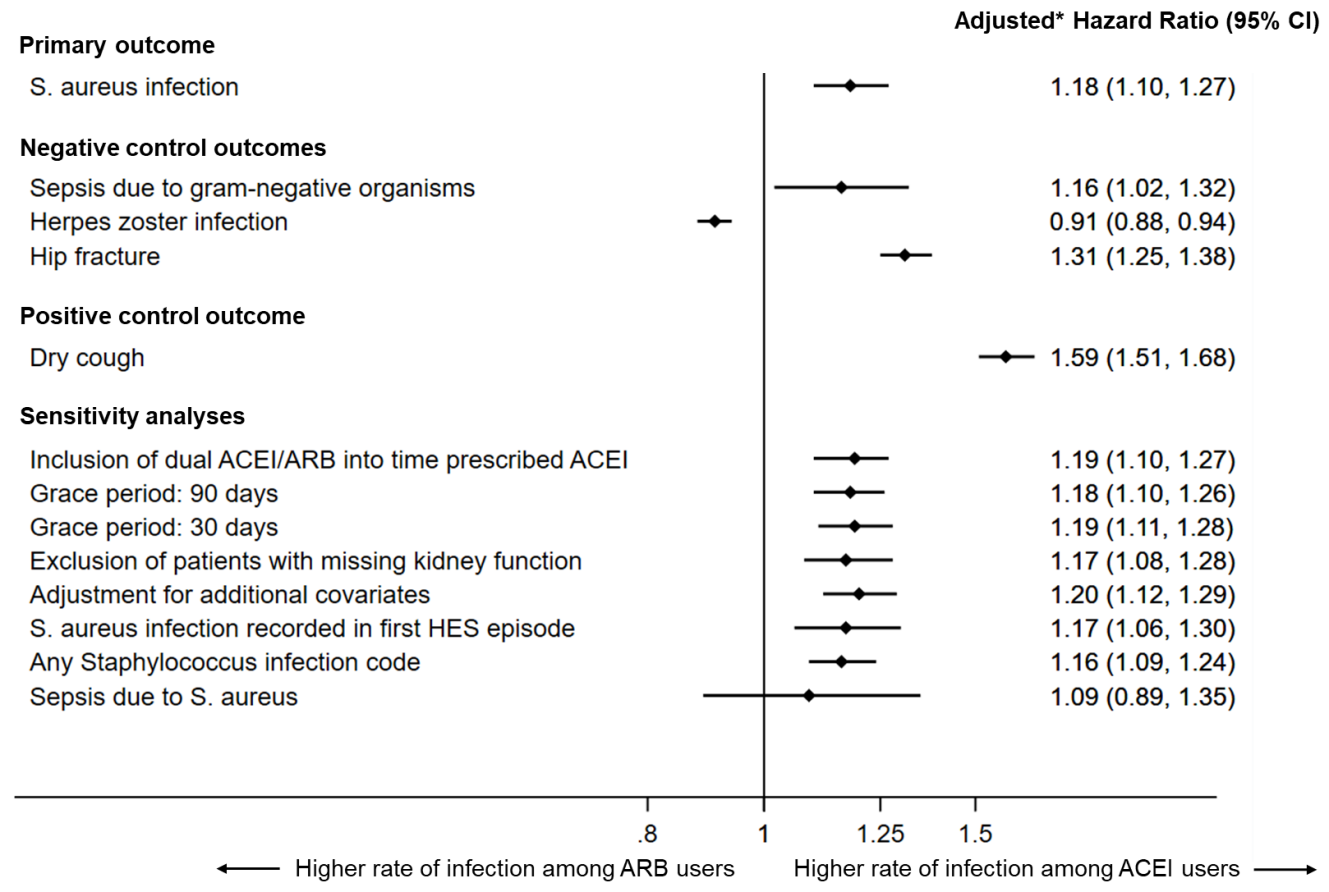
Forest plot of fully adjusted hazard ratios for the outcomes for all analyses, comparing rates during periods of ACEI and ARB prescription. *Adjusted for age (<55, 55–64, 65–74, 75–84, and ≥85 years), sex, year (1997–2001, 2002–2006, 2007–2011, 2012–2016), kidney function (eGFR ≥60 mL/min/1.73m ^2^ or unmeasured, eGFR 45–59 mL/min/1.73m ^2^, eGFR 30–44 mL/min/1.73m ^2^, eGFR <30 mL/min/1.73m ^2^, or renal replacement therapy), diagnoses of hypertension, diabetes, myocardial infarction, heart failure, and proteinuria: all covariates were time-updated.

In the main analysis, the adjusted hazard ratio (HR) for the first hospitalisations with infection due to *S. aureus* comparing periods with ACEI vs ARB prescription was 1.18 (95% CI 1.10 – 1.27) ( Table 2; Figure 2). For the control analyses, we also found an association between ACEI prescription and sepsis due to Gram-negative organisms (adjusted HR 1.16, 95% CI 1.02 – 1.32), hip fracture (adjusted HR 1.31, 95% CI 1.25 – 1.38) and dry cough (adjusted HR 1.59, 95% CI 1.51 – 1.68) while risk of herpes zoster was lower among users of ACEI (adjusted HR 0.91, 95% CI 0.88 – 0.94).

Sensitivity analyses showed similar results ( Figure 2; Supplementary Table 2, *Extended data* ^19^), except that the strength of association between ACEI and sepsis due to S. aureus was smaller (adjusted HR 1.09, 95% CI 0.89 – 1.35). In subgroup analyses, there were no clear differences in the adjusted hazard ratio (HR) for rates of *S. aureus* infection by year and smoking status ( **Supplementary Table 3**, *Extended data* ^19^).

## Discussion

Our study demonstrated that rates of *S. aureus* infection are higher among users of ACEI than users of ARBs, consistent with the hypothesis from animal models we sought to examine ^6^. However, our pre-planned control analysis where we did not expect to see an association, rates of Gram-negative sepsis, was also more common in ACEI users. This could suggest that taking ACEI leads to an increased susceptibility to infection by both types of organism, or that the association with drug class is due to underlying differences between users of ACEI and ARB that remained despite adjustment for potential confounders.

The importance of this question given the potential role of ACEI and ARB drugs in leading to severe outcomes in people with COVID-19 infection led to us undertaking *post-hoc* control analyses to explore further the possibility of residual confounding. For these analyses we did not expect choice of ACEI or ARB to be causally associated with the outcome but found that rates of hip fracture were higher in people taking ACEI, while herpes zoster infection was higher among those taking ARBs. The positive control, rate of dry cough, was substantially higher among ACEI users as we expected, demonstrating that our approach was able to detect known drug effects. Overall, the additional analyses suggest that the associations of ACEI use with *S. aureus* and Gram-negative sepsis is due to confounding rather than the drugs causing susceptibility to infection.

Comparison of the unadjusted and adjusted hazard ratios for analyses of *S. aureus* and Gram-negative sepsis, shows that the adjusted results move towards the null. This suggests that some of the increased rate of infections in ACEI users is due to factors that are associated with drug prescription such as prevalence of diabetes and sex, and taking this into account reduces the difference in rates between the drug classes. In this analysis we had pre-selected covariates with the aim of trying to capture confounding as well as possible given the data source. However, even adjustment for multiple other potential confounders in our sensitivity analysis did not reduce the hazard ratio substantially closer to the null.

By contrast with our other analyses, rates of herpes zoster infection were higher in ARB users. Confounder adjustment moved the hazard ratio closer to the null but residual confounding remained. This may be because strong risk factors for infection such as conditions associated with immunosuppression were not included in our analysis, as well as family history which is hard to define in routine data sources ^24^. Hip fracture was more common in ACEI users but confounder adjustment moved the hazard ratio away from the null suggesting negative confounding. This may be because women were more likely to be prescribed ARBs, and female sex is a major risk factor for hip fracture ^25^.

The pre-planned sensitivity analyses altering our definition of drug exposure, covariate adjustment or outcome all showed results very similar to the main analysis suggesting that the changes did not substantially affect residual confounding. We undertook pre-planned stratified analyses to explore potential sources of confounding. We examined the association within shorter time intervals over the study period which demonstrated consistent results over the last 15 years, suggesting that focus on detection of Staphylococcal infection related to efforts to reduce rates of methicillin-resistant *S. aureus* (MRSA) did not impact our results ^26^. Similarly, we considered misclassification of ex-smokers as non-smokers to be a potential source of information bias potentially contributing to undetected poorer health status among users of ACEI in the analysis and partly explaining our results. Interestingly, in stratified analyses the highest adjusted HR was seen among non-smokers suggesting that this consideration should be explored further, but confidence intervals overlapped so this result should be interpreted with caution.

We have used time-updated drug exposure which is vital for exploring causal relationships since we show that approximately 20% of initiators in each class switch from ACEI to ARB or vice versa and thus in a cohort study would be misclassified based on baseline drug use. In addition, our primary analysis compares outcomes during time exposed to ACEI to that exposed to ARB. However, to highlight the implications of study design for future COVID-19 related studies, we chose to present the rates of outcomes during time exposed to neither drug ( Table 2). These results show that risk of all outcomes except herpes zoster is markedly greater during this ‘unexposed’ time. We propose that this is because the drugs are stopped during periods of illness or increasing frailty, and hence unexposed time after drug initiation is associated with a greater risk of adverse outcomes. Inclusion of these time periods in analyses based on baseline drug exposure may lead to inappropriate conclusions.

There are a number of strengths in our analysis. We have conducted a large cohort study using a data source with detailed information on confounders and linkage to hospital admission data. We have used a range of control and stratified analyses to explore the possibility and sources of residual confounding. However, we have adjusted for the same covariates for all analyses while the underlying confounding structure may be different. Nonetheless, adjustment for additional covariates in our sensitivity analyses made no important difference to the main analysis. Additional adjustment using techniques such as high-dimensional propensity scores may reduce confounding compared to standard multivariable regression ^27^. There is limited validation of the codes used to define outcomes in these analyses, but there is no reason to think their recording would differ by ACEI or ARB use.

A number of influential population-based epidemiological analyses have compared outcomes between ACEI and ARB users ^7– 10^. Some of these have used considerations such as stratified analysis by cumulative duration of drug use, and by time since initiation to examine outcomes including dementia and cancer, concluding that associations are unlikely to be causal ^8, 9^. However, these approaches are less relevant to acute outcomes, such as infection risks, which can occur at any stage in treatment.

ACEI and ARB drugs are prescribed to millions of people globally making a potential association with severity of COVID-19 an issue of substantial public health importance. Population based epidemiological studies may help to address this issue. However, subtle differences in the characteristics of people prescribed ACEI and ARB, those prescribed ACEI or ARB for different indications and people prescribed ACEI or ARB compared to other antihypertensives can lead to non-causal associations in studies of drug effects. Recommendations about cessation or use of these important and evidence-based medications in relation to COVID-19 infection of these drugs may require completion of randomised clinical trials which are currently underway ^14, 28^.

## Conclusion

Using primary care data in the UK, rates of infection with *S. aureus* are higher among users of ACEI compared to ARBs. However, other control outcomes also show an association with drug class, with similar or greater effect size. This suggests that there are sources of residual confounding in comparisons between users of ACEI and ARBs. Observational analyses studying potential causal associations of outcomes with ACEI or ARB drugs, such as in relation to COVID-19 infection, should be conducted and interpreted with caution.

## Data availability

### Underlying data

The datasets used for these analyses are not publicly available due to Clinical Practice Research Datalink (CPRD) licensing restrictions. Data access through CPRD is subject to protocol approval by an Independent Scientific Advisory Committee (ISAC). All protocols must be submitted to the ISAC Secretariat using the Protocol Application Form. The protocol application form and where to submit the form are detailed on the CPRD website: https://cprd.com/research-applications.

LSHTM Data Compass: Codelist for: "Comparisons of Staphylococcus aureus infection and other outcomes between users of angiotensin-converting-enzyme inhibitors and angiotensin II receptor blockers: lessons for COVID-19 from a nationwide cohort study", https://doi.org/10.17037/data.00001668 ^20^

Data are available under the terms of the Creative Commons Attribution 4.0 International license (CC-BY 4.0).

### Extended data

LSHTM Research Online: Supplementary data for “Comparisons of Staphylococcus aureus infection and other outcomes between users of angiotensin-converting-enzyme inhibitors and angiotensin II receptor blockers: lessons for COVID-19 from a nationwide cohort study”, https://doi.org/10.17037/PUBS.04656578 ^19^

This project contains the following extended data:

- Supplementary Figure 1. Diagram of study design with definition of exposure periods.
- Supplementary Table 1. Characteristics of the 487,165 study participants during follow-up.
- Supplementary Table 2. Crude rates and adjusted hazard ratio for the incidence of Staphylococcus aureus infections comparing periods prescribed angiotensin-converting-enzyme inhibitors and angiotensin II receptor blockers.
- Supplementary Table 3. Crude rates and adjusted hazard ratio for the incidence of Staphylococcus aureus infection, comparing periods prescribed angiotensin-converting-enzyme inhibitors and angiotensin II receptor blockers, stratified by calendar period and smoking status.

Data are available under the terms of the Creative Commons Attribution 3.0 International license (CC-BY 3.0).
