# Supplementary material for: Comparisons of Staphylococcus aureus infection and other outcomes between users of angiotensin-converting-enzyme inhibitors and angiotensin II receptor blockers: lessons for COVID-19 from a nationwide cohort study
Source: Wellcome Open Res. 2020 Apr 27;5:77. [Version 1] doi: 10.12688/wellcomeopenres.15873.1 (PMC7262569; doi:10.12688/wellcomeopenres.15873.1)
Supplement: Peer review history

# Peer review: Reviewer response for version 1

**DOI:** 10.21956/wellcomeopenres.17410.r38729

**Published:** 2020-05-29

**Authors:** Chien-Chang Lee, Wan-Ting Hsu

**Affiliations:** 1 Department of Epidemiology, Harvard T.H. Chan School of Public Health, Boston, MA, USA; 2 Department of Emergency Medicine, National Taiwan University Hospital, Taipei, Taiwan; 3 Center of Intelligent Healthcare, National Taiwan University Hospital, Taipei, Taiwan

The authors used an exposure crossover design to investigate whether ACEI use is associated with *Staphylococcus aureus* infection as compared with ARB use. This is a strong design with multiple sensitivity analyses. The authors shown a good epidemiologic justification for the study. Overall the paper is well written and contributes to our understanding on this topic. My comments are as follows. I agree that ACEI/ARB use is an important topic in COVID-19 research. However, the authors may need to tone down “lessons for COVID-19 from a nationwide cohort study” in the title. They may not over-interpret their design and results to implicate COVID-19. *Staphylococcus aureus* infection is different from viral infection. I would recommend avoiding speculations on COVID-19 infection; although timely, all data were collected before the pandemics.

 They may consider to discuss and cite the following paper: Hsu *et al. *(2020) ^1^.

Is the work clearly and accurately presented and does it cite the current literature?

Yes

If applicable, is the statistical analysis and its interpretation appropriate?

Yes

Are all the source data underlying the results available to ensure full reproducibility?

Yes

Is the study design appropriate and is the work technically sound?

Yes

Are the conclusions drawn adequately supported by the results?

Yes

Are sufficient details of methods and analysis provided to allow replication by others?

Yes

Reviewer Expertise:

Pharmacoepidemiology, Sepsis, Infectious disease, Biomarker, Translational diagnostic science, Big data epidemiology, Hypertension.

We confirm that we have read this submission and believe that we have an appropriate level of expertise to confirm that it is of an acceptable scientific standard.

## References

[1] : Effect of Renin-Angiotensin-Aldosterone System Inhibitors on Short-Term Mortality After Sepsis: A Population-Based Cohort Study. *Hypertension*.2020;75(2) : 10.1161/HYPERTENSIONAHA.119.13197 483-491 10.1161/HYPERTENSIONAHA.119.13197 31838905

# Response

**Published:** 2020-05-29

**Authors:** Laurie Tomlinson

**Affiliations:** LSHTM, UK

We thank the reviewers very much for their review, and for drawing our attention to their relevant paper. We agree that we must be cautious about extrapolating our results to the current pandemic: however our attention with the paper was merely to discuss potential epidemiological pitfalls and considerations related to comparisons of these drugs in future work. Therefore the aim of the title was to highlight its relevance in this time when information needs to be accessed quickly.

# Peer review: Reviewer response for version 1

**DOI:** 10.21956/wellcomeopenres.17410.r38536

**Published:** 2020-05-20

**Authors:** Daniel Morales

**Affiliations:** 1 Population Health and Genomics Division, University of Dundee, Dundee, UK

This is a well written article and robust study examining the association between ACEI use and the risk of Staph aureus infections compared with exposure to ARBs. The study uses a large cohort of patients identified from a well-established data source used for research. Such large data sources allow relatively small associations to be identified that are statistically significant and these may raise public health concern. For this reason, evaluating causality is very important.

 Despite the underlying biological mechanism seen in animal studies the results of this study suggest that the statistically significant association observed between ACEI use and Staph aureus infections is likely to be non-causal. This is supported by the finding of significant associations with several negative controls.

 It is interesting to observe the stronger associations during unexposed periods of ACEI and ARB therapy i.e. when withheld or withdrawn. I agree with the interpretation of the authors that this is likely to be in keeping with patients being sicker or frailer during these spells. There could also be a contributing role of prolonged periods of hospitalisation when routine prescriptions issued by the GP may not occur.

 We are not completely sure therefore why this association is observed. Whilst unobserved confounders may exist, significant associations remained robust despite adjustment with several addiitonal clinical covariates. Whilst similar proportions of new ACE and new ARB users ultimately switch, far more patients are new users of ACEI rather than new users of ARBs. Therefore much more person time exposed to an ARB may result from having switched rather than being a new user of ARB. It is uncertain what proportion of ARB person time results from being a new user as opposed to a switcher, and whether this type of difference might be one source of the residual unmeasured confounding.

 The supplementary material is very informative. As it is not too extensive it could be useful to deposit this together with the main article so that everything is located in one place.

 Overall, this study is a useful example of why it is important to test the robustness of significant associations in observational studies before making recommendations to change clinical practice.

Is the work clearly and accurately presented and does it cite the current literature?

Yes

If applicable, is the statistical analysis and its interpretation appropriate?

Yes

Are all the source data underlying the results available to ensure full reproducibility?

Yes

Is the study design appropriate and is the work technically sound?

Yes

Are the conclusions drawn adequately supported by the results?

Yes

Are sufficient details of methods and analysis provided to allow replication by others?

Yes

Reviewer Expertise:

Pharmacoepidemiology and primary care.

I confirm that I have read this submission and believe that I have an appropriate level of expertise to confirm that it is of an acceptable scientific standard.
